# Supplementary material for: Ketamine increases activity of a fronto-striatal projection that regulates compulsive behavior in SAPAP3 knockout mice
Source: Nat Commun. 2021 Oct 15;12:6040. doi: 10.1038/s41467-021-26247-2 (PMC8519915; doi:10.1038/s41467-021-26247-2)
Supplement: Supplementary file 1 — Supplementary Information [file 41467_2021_26247_MOESM1_ESM.pdf]

Supplementary Information

Davis et al Supplementary Figure 1

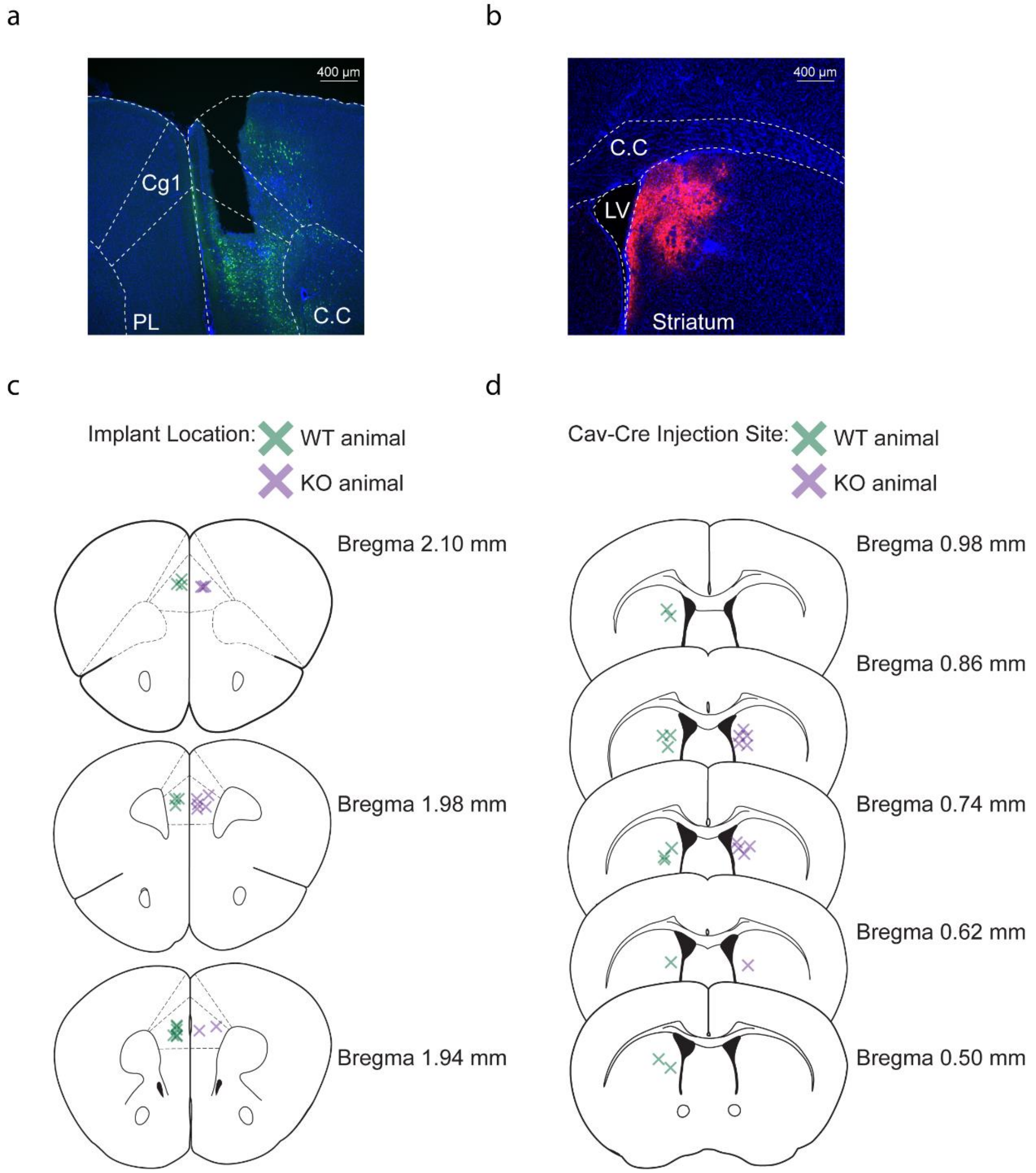

Supplementary Figure 1. Implant location and viral injection sites for fiber photometry brains. (a) Representative image of implant location with neurons (in green) expressing AAV1.Syn.Flex.GCaMP6m.WPRE.SV40 (Cg1 = cingulate cortex, PL = prelimbic, C.C = corpus callosum, LV = lateral ventricle). **(b)** Representative image of viral injection site of 50/50 mixture of AAV1.hSyn.mCherry (to visualize injection location) and CAV2-Cre. **(c)** Mapping of implant location in the dmPFC. WT animals (N = 11) are marked on the left hemisphere with green x's. KO animals (N = 10) are marked on the right hemisphere with purple x's. **(d)** Mapping of CAV2-cre injection sites in the striatum. WT animals (N = 11) are marked on the left hemisphere with green x's. KO animals (N = 10) are marked on the right hemisphere with purple x's.

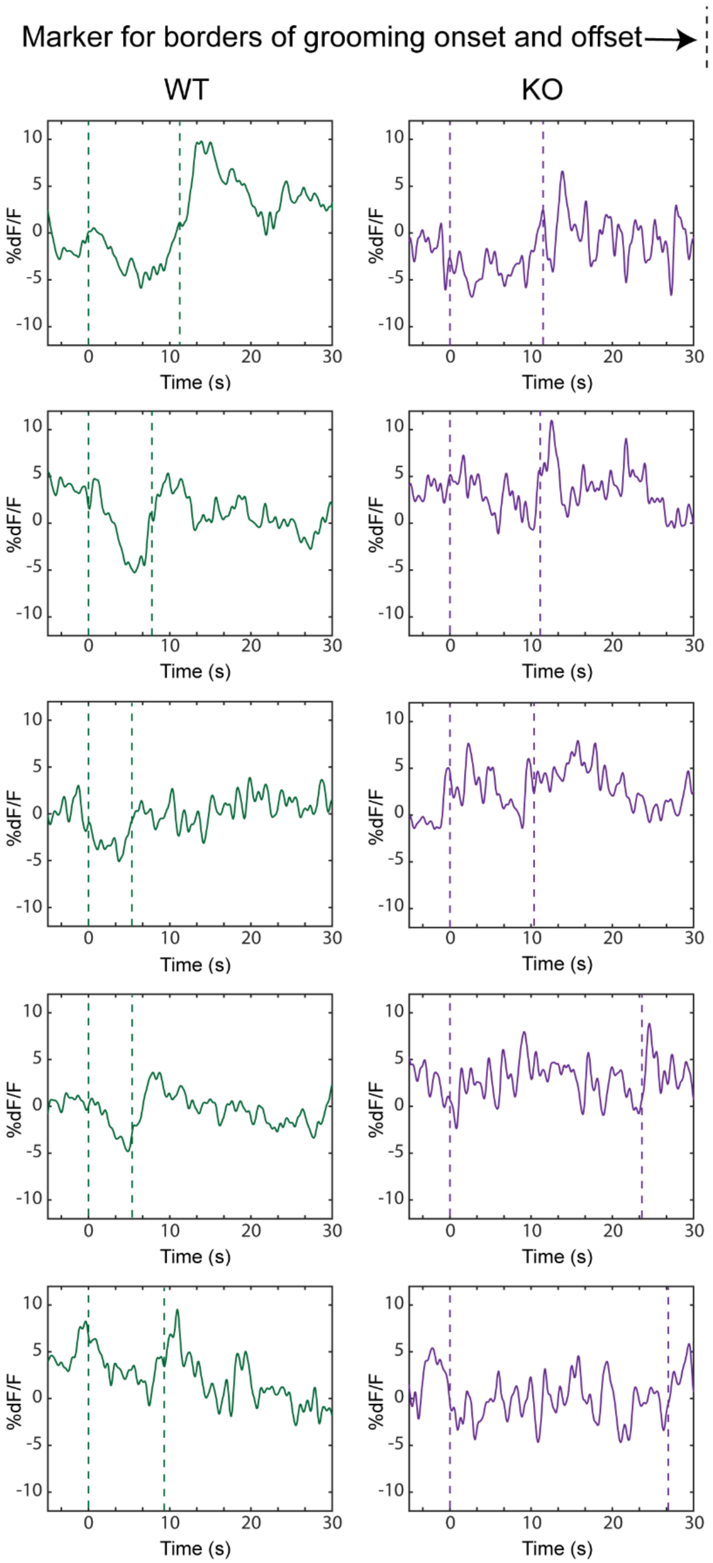

Supplementary Figure 2. Raw fiber photometry traces showing full grooming bouts. Beginning of grooming bouts are marked by a dashed line at the 0 second timepoint and end of grooming bouts are marked by the second dashed ling. Examples traces of full grooming bouts from WT mice (green traces) and SAPAP3 KO mice (purple traces) are contained within the boundaries of the dashed lines.

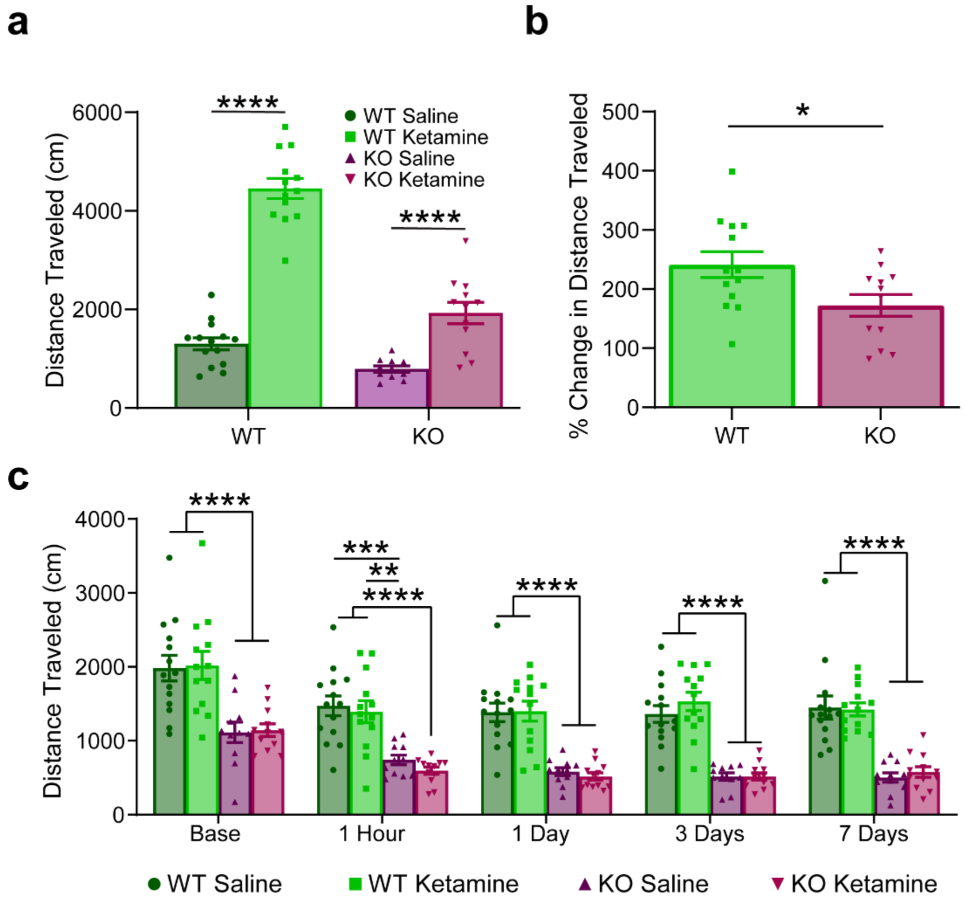

Supplementary Figure 3. Locomotor effects of ketamine on SAPAP3 KO and WT mice. Bar and line graphs show data means +/- SEM. * = P <0.05, ** = P < 0.01, *** = P < 0.001, **** = P < 0.0001. **(a)** Both WT and KO mice treated with ketamine show a significant acute increase in locomotor activity 10 minutes post-injection (Two-way ANOVA: interaction P < 0.0001, df = 1, F = 36.88; genotype P < 0.0001, df = 1, F = 83.34; experimental group P < 0.0001, df = 1, F = 166.2, residual df = 46; Sidak's multiple comparisons: WT saline vs WT ketamine P <0.0001, KO saline vs KO ketamine P <0.0001; WT-saline = 14, KO-saline = 11, WT-ketamine = 13, KO-ketamine = 12). **(b)** Ketamine induces a larger increase in locomotor behavior (over 200%) in WTs (N = 13) compared to KOs (N = 12), when ketamine-induced locomotor behavior is normalized to baseline locomotor behavior measures (unpaired two-tailed t-test P = 0.0250, df = 23). **(c)** Locomotor activity was recorded across the same experimental time window as grooming in Figure 1. KO mice, regardless of treatment, move significantly less than WT littermates (Two-way RM ANOVA: interaction P = 0.8027, df = 12, F = 0.6438; day P < 0.0001, df = 4, F = 42.76; experimental group P < 0.0001, df = 3, F = 27.11, residual df = 184; Tukey's multiple comparisons: Base WT Saline vs. KO Saline P < 0.0001, WT Saline vs. KO Ketamine P < 0.0001, WT Ketamine vs. KO Saline P < 0.0001, WT Ketamine vs. KO Ketamine P < 0.0001; 1 hour WT

Saline vs. KO Saline P = 0.001, WT Saline vs. KO Ketamine P < 0.0001, WT Ketamine vs. KO Saline P = 0.0010, WT Ketamine vs. KO Ketamine P < 0.0001; 1 day WT Saline vs. KO Saline P < 0.0001, WT Saline vs. KO Ketamine P < 0.0001, WT Ketamine vs. KO Saline P < 0.0001, WT Ketamine vs. KO Ketamine P < 0.0001; 3 days WT Saline vs. KO Saline P < 0.0001, WT Saline vs. KO Ketamine P < 0.0001, WT Ketamine vs. KO Saline P < 0.0001, WT Ketamine vs. KO Ketamine P < 0.0001; 7 days WT Saline vs. KO Saline P < 0.0001, WT Saline vs. KO Ketamine P < 0.0001, WT Ketamine vs. KO Saline P < 0.0001, WT Ketamine vs. KO Ketamine P < 0.0001; WT-saline = 14, KO-saline = 11, WT-ketamine = 13, KO-ketamine = 12).

Davis et al Supplementary Figure 4

Figure 2b grooming frequency			
Tukey's multiple comparisons test	95.00% CI of diff.	Summary	Adjusted P Value
BASE			
WT Saline vs. WT Ketamine	-11.08 to 11.89	ns	0.9997
WT Saline vs. KO Saline	-27.48 to -3.453	**	0.0055
WT Saline vs. KO Ketamine	-31.77 to -8.305	****	<0.0001
WT Ketamine vs. KO Saline	-28.09 to -3.658	**	0.005
WT Ketamine vs. KO Ketamine	-32.38 to -8.505	****	<0.0001
KO Saline vs. KO Ketamine	-17.02 to 7.879	ns	0.7779
1 HOUR			
WT Saline vs. WT Ketamine	-12.50 to 10.47	ns	0.9958
WT Saline vs. KO Saline	-40.62 to -16.59	****	<0.0001
WT Saline vs. KO Ketamine	-15.85 to 7.612	ns	0.8002
WT Ketamine vs. KO Saline	-39.80 to -15.37	****	<0.0001
WT Ketamine vs. KO Ketamine	-15.04 to 8.835	ns	0.9074
KO Saline vs. KO Ketamine	12.04 to 36.93	****	<0.0001
1 DAY			
WT Saline vs. WT Ketamine	-11.17 to 11.80	ns	0.9999
WT Saline vs. KO Saline	-36.43 to -12.40	****	<0.0001
WT Saline vs. KO Ketamine	-20.29 to 3.171	ns	0.2359
WT Ketamine vs. KO Saline	-36.95 to -12.52	****	<0.0001
WT Ketamine vs. KO Ketamine	-20.82 to 3.059	ns	0.2205
KO Saline vs. KO Ketamine	3.409 to 28.30	**	0.0062
3 DAY			
WT Saline vs. WT Ketamine	-13.28 to 9.694	ns	0.9777
WT Saline vs. KO Saline	-33.18 to -9.154	****	<0.0001
WT Saline vs. KO Ketamine	-28.36 to -4.900	**	0.0017
WT Ketamine vs. KO Saline	-31.59 to -7.162	***	0.0003
WT Ketamine vs. KO Ketamine	-26.78 to -2.903	**	0.008
KO Saline vs. KO Ketamine	-7.909 to 16.99	ns	0.7814
7 DAY			
WT Saline vs. WT Ketamine	-10.30 to 12.67	ns	0.9933
WT Saline vs. KO Saline	-23.62 to 0.4040	ns	0.0624
WT Saline vs. KO Ketamine	-22.16 to 1.302	ns	0.1009
WT Ketamine vs. KO Saline	-25.01 to -0.5812	*	0.036
WT Ketamine vs. KO Ketamine	-23.55 to 0.3217	ns	0.0598
KO Saline vs. KO Ketamine	-11.27 to 13.63	ns	0.9948
Figure 2e grooming duration			
Tukey's multiple comparisons test	95.00% CI of diff.	Summary	Adjusted P Value
BASE			
Saline KO vs. Ketamine KO	-92.91 to 19.48	ns	0.3309
Saline KO vs. Saline WT	-3.466 to 105.0	ns	0.0757
Saline KO vs. Ketamine WT	-5.430 to 104.9	ns	0.0936
Ketamine KO vs. Saline WT	34.53 to 140.5	***	0.0002
Ketamine KO vs. Ketamine WT	32.54 to 140.3	***	0.0003
Saline WT vs. Ketamine WT	-52.91 to 50.80	ns	>0.9999
1 HOUR			
Saline KO vs. Ketamine KO	-7.740 to 104.7	ns	0.1178
Saline KO vs. Saline WT	27.75 to 136.2	***	0.0007
Saline KO vs. Ketamine WT	17.66 to 128.0	**	0.0041
Ketamine KO vs. Saline WT	-19.43 to 86.50	ns	0.3591
Ketamine KO vs. Ketamine WT	-29.54 to 78.25	ns	0.6467
Saline WT vs. Ketamine WT	-61.03 to 42.68	ns	0.968
1 DAY			
Saline KO vs. Ketamine KO	-18.89 to 93.51	ns	0.3167
Saline KO vs. Saline WT	37.37 to 145.9	***	0.0001
Saline KO vs. Ketamine WT	34.91 to 145.2	***	0.0002
Ketamine KO vs. Saline WT	1.343 to 107.3	*	0.0421
Ketamine KO vs. Ketamine WT	-1.139 to 106.7	ns	0.0575
Saline WT vs. Ketamine WT	-53.41 to 50.30	ns	0.9998
3 DAY			
Saline KO vs. Ketamine KO	-51.09 to 61.30	ns	0.9954
Saline KO vs. Saline WT	12.17 to 120.7	**	0.0094
Saline KO vs. Ketamine WT	8.999 to 119.3	*	0.0153
Ketamine KO vs. Saline WT	8.344 to 114.3	*	0.016
Ketamine KO vs. Ketamine WT	5.154 to 112.9	*	0.0255
Saline WT vs. Ketamine WT	-54.11 to 49.60	ns	0.9995
7 DAY			
Saline KO vs. Ketamine KO	-91.26 to 21.14	ns	0.3725
Saline KO vs. Saline WT	-4.401 to 104.1	ns	0.0842
Saline KO vs. Ketamine WT	-3.611 to 106.7	ns	0.0764
Ketamine KO vs. Saline WT	31.94 to 137.9	***	0.0003
Ketamine KO vs. Ketamine WT	32.71 to 140.5	***	0.0003
Saline WT vs. Ketamine WT	-50.16 to 53.55	ns	0.9998

Supplementary Figure 4. Exact P values from multiple comparisons on datasets from Figure 2b/2e.

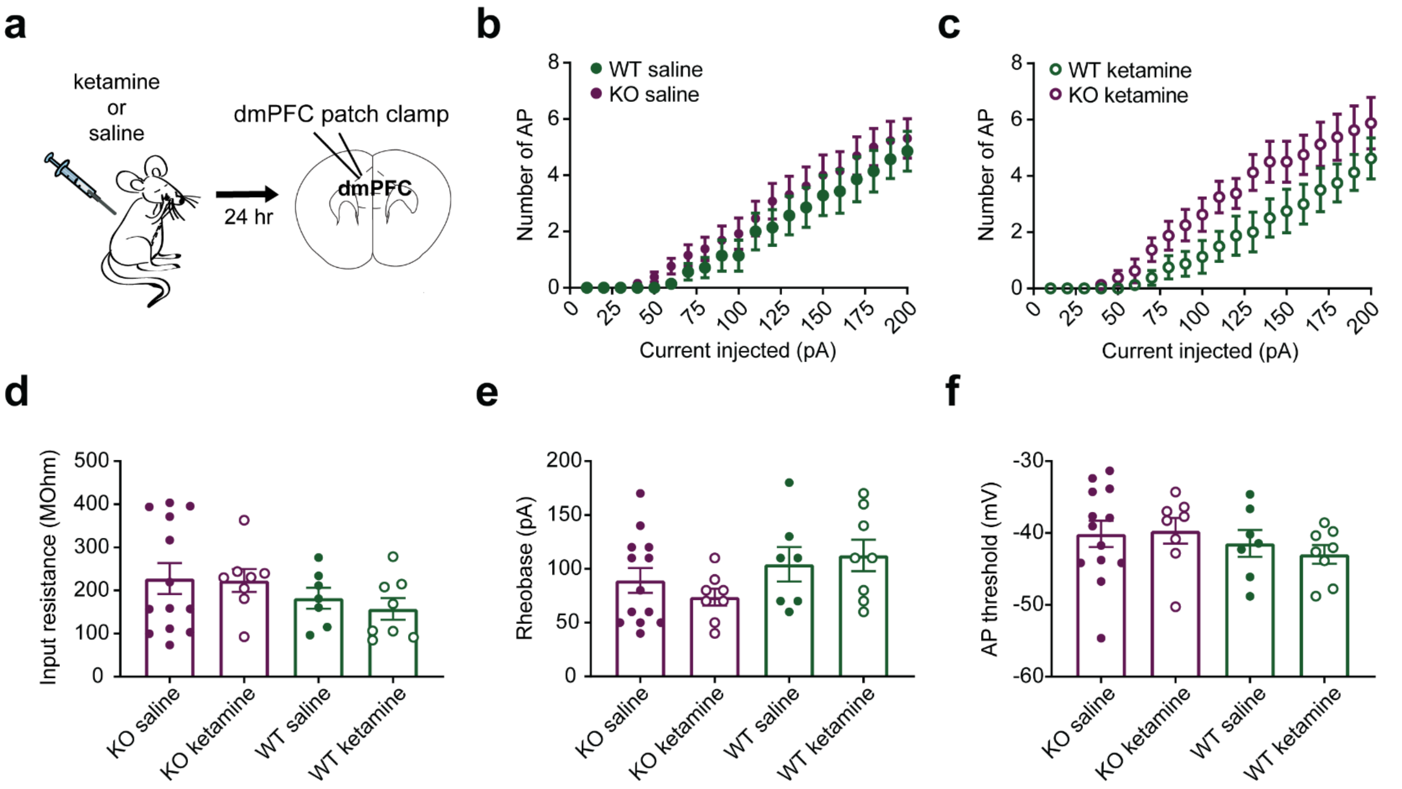

Supplementary Figure 5. Effects of ketamine and genotype on intrinsic dmPFC cell properties. Bar and line graphs show data means +/- SEM **(a)** Experimental schematic of i.p. injections followed by slice recordings 24 hours later (WT saline N = 7 cells, KO saline N = 13 cells, WT ketamine N = 8 cells, KO ketamine N = 8 cells). **(b)** Input-output curves compared between WT saline and KO saline did not reveal any group differences, just a main effect of sequential current injected (Two-way RM ANOVA: interaction P = 0.9933, df = 19, F = 0.3720; current injected P < 0.0001, df = 19, F = 55.91; experimental group P = 0.4336, df = 1, F = 0.6415, residual df = 342). **(c)** Input-output curves generated from WT ketamine and KO ketamine (30 mg/kg) groups. There is a main effect of current injected and a main interaction between current injected and experimental group, indicating that ketamine could be having different effects on the input-output curves of KOs compared to WTs, but no further effects were seen with multiple comparisons (Two-way RM ANOVA: interaction P = 0.0031, df = 19, F = 2.210; current injected P < 0.0001, df = 19, F = 57.89; experimental group P = 0.0930, df = 1, F = 3.249, residual df = 266). No difference was seen across genotype or treatment condition for **(d)** input resistance (Kruskal-Wallis test P = 0.4435) **(e)** rheobase (Kruskal-Wallis test P = 0.2652) or **(f)** action potential (AP) threshold (Kruskal-Wallis test P = 0.4370).

Davis et al Supplementary Figure 6

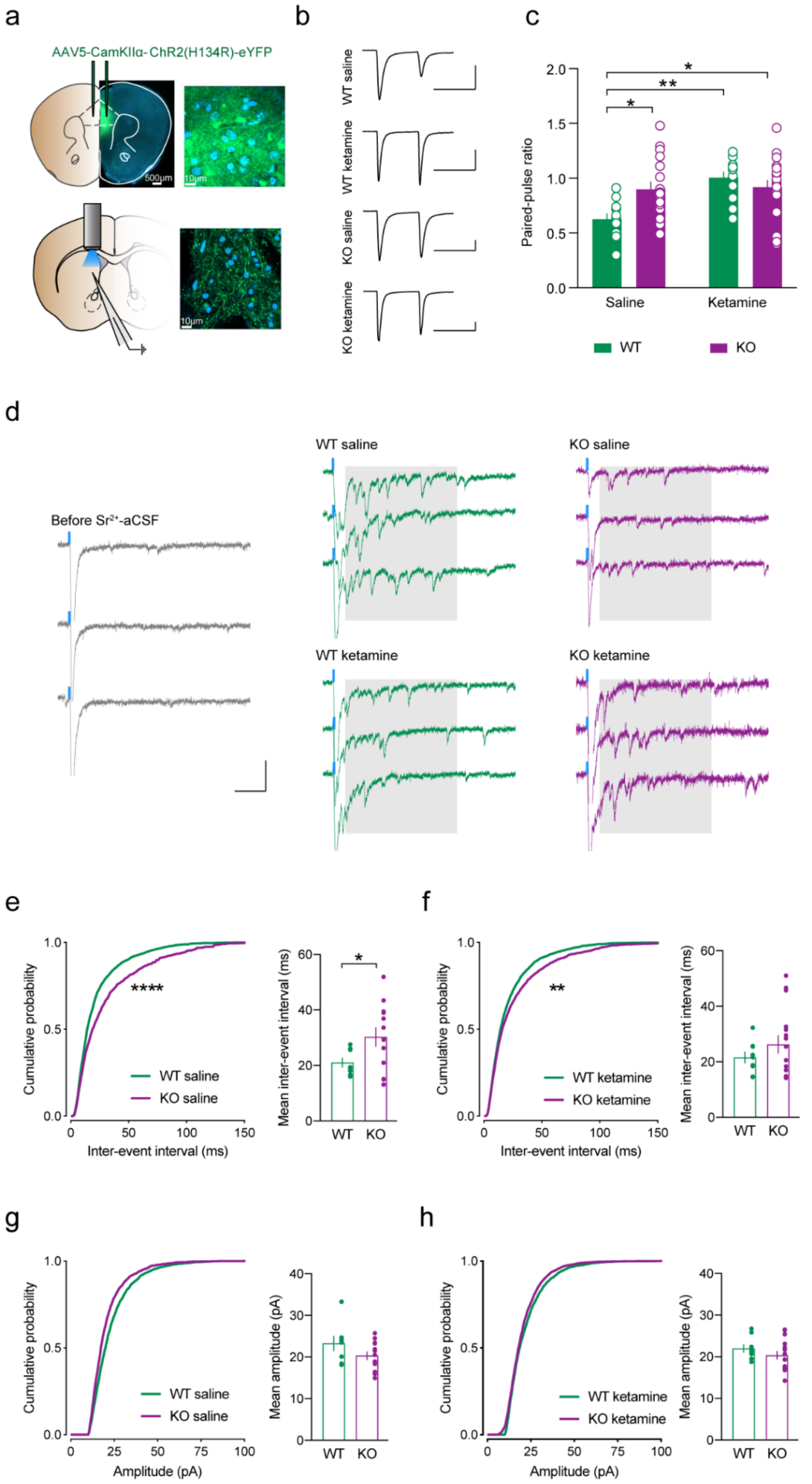

Supplementary Figure 6. Effects of ketamine and genotype on dmPFC-DMS synaptic properties. (a) (A) Experimental details. Mice were injected a virus in the dmPFC to express ChR2(H134R) (top left). ChR2(H134R) in the dmPFC (green, top right). Patch-clamp slice electrophysiology was conducted in the DMS (bottom left), where dmPFC terminals expressed ChR2(H134R) (green, bottom right). For representative images, cell nuclei were stained with Hoechst (blue). (B) Example traces of patch-clamp recordings to determine paired-pulse ratios. Scale bars are 50 ms, 200 pA. (C) Paired-pulse ratios from dmPFC-DMS synapses in WT and KO mice that were treated with saline or ketamine (30 mg/kg, i.p.) 24 h before sacrifice. Two-way ANOVA with significant interaction ($P = 0.0050$, $F(1, 58) = 8.532$) and main treatment effect ($P = 0.0023$, $F(1, 58) = 10.19$). Sidak post-hoc test: *P = 0.0195 (WT saline vs. KO saline); *P = 0.0102 (WT saline vs. KO ketamine); **P = 0.0013 (WT saline vs. WT ketamine). N (cells) = 12 (WT saline), 18 (KO saline), 13 (WT ketamine), 19 (KO ketamine). (D) Example traces from patch-clamp recordings in strontium-containing aCSF (Sr2+-aCSF) for asynchronous release. Three consecutive traces from a saline-treated WT recorded in normal aCSF (left), and from recordings in Sr2+-aCSF (all conditions; middle and right). Grey shading indicates window of analysis. Blue line indicates blue-light stimulation of dmPFC-DMS terminals. Scale bar is 50 ms, 50 pA. (E) Left: inter-event interval cumulative probability of dmPFC-DMS asynchronous release from saline-treated WT vs. KO mice. Two-sample Kolmogorov-Smirnov test, ****P = 3.1 x 10-7. Right: Mean inter-infusion interval from saline-treated WT vs. KO mice. Two-tailed unpaired t-test with Welch's correction, *P = 0.023, t=2.495, df=16.85. (F) Left: inter-event interval cumulative probability of dmPFC-DMS asynchronous release from ketamine-treated WT vs. KO mice. Two-sample Kolmogorov-Smirnov test, **P = 1.6 x 10-3. Right: mean inter-infusion interval from ketamine-treated WT vs. KO mice. Two-tailed unpaired t-test with Welch's correction, P = 0.21, t=1.282, df=22.43. (G) Left: amplitude cumulative probability of dmPFC-DMS asynchronous release from saline-treated WT vs. KO mice. Two-sample Kolmogorov-Smirnov test was not significant (P = 0.22). Right: mean amplitude from saline-treated WT vs. KO mice. Two-tailed unpaired t-test, P = 0.12, t=1.649, df=19. (H) Left: amplitude cumulative probability of dmPFC-DMS asynchronous release from ketamine-treated WT vs. KO mice. Two-sample Kolmogorov-Smirnov test was not significant (P = 0.14). Right: mean amplitude from ketamine-treated WT vs. KO mice. Non-significant two-tailed unpaired t-test, P = 0.26, t=1.166, df=23. N (cells) = 8 (WT saline), 13 (KO saline), 9 (WT ketamine), 16 (KO ketamine) for data in E – H. Data are mean ± SEM.

Davis et al Supplementary Figure 7

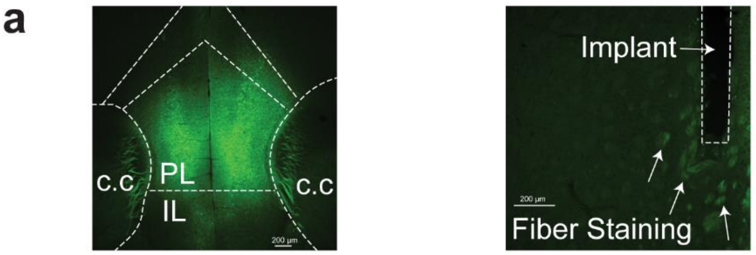

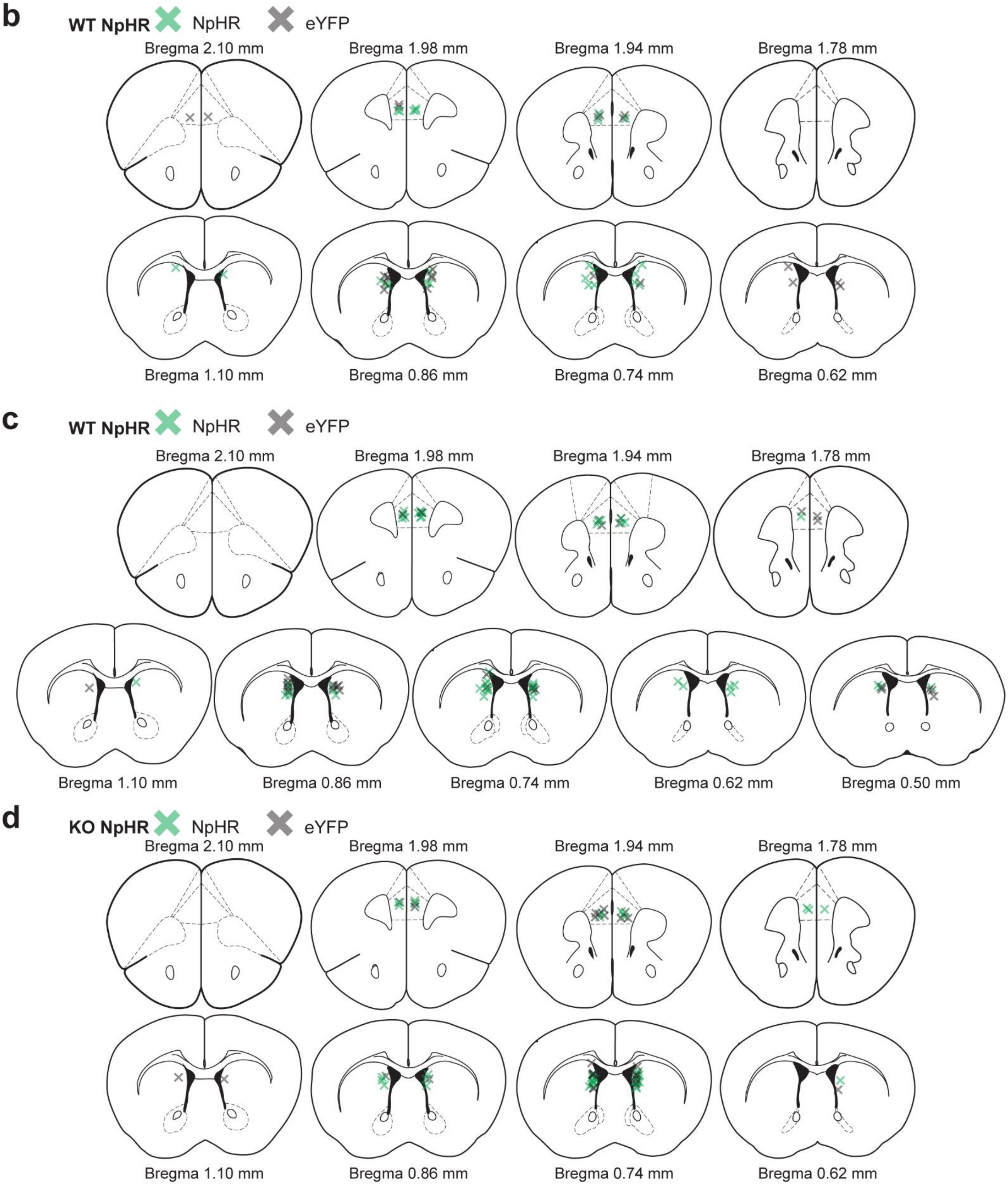

Supplementary Figure 7. NpHR experiments histology (a) Representative images of AAV5.EF1a.DIO.eNpHR3.0-eYFP viral expression (left) and implant site (right, white arrows highlight fiber staining) (IL = infralimbic cortex, PL = prelimbic cortex, c.c = corpus callosum). **(b)** For the open-loop inhibition experiment, mapping of viral injection sites in the dmPFC (top row of brain slices) and DMS implant sites (bottom row of brain slices) for WT mice expressing either eNpHR3.0 (green x's) or eYFP (grey x's). WT animals (N = 8/group). Demarcation of viral injection sites for dmPFC is represented by only one x in one hemisphere but injections were bilateral. **(c)** For the closed-loop inhibition experiment, mapping of viral injection sites in the dmPFC (top row of brain slices) and DMS implant sites (bottom row of brain slices) for WT mice expressing either eNpHR3.0 (green x's, N = 18) or eYFP (grey x's, N = 10). Demarcation of viral injection sites for dmPFC is represented by only one x in one hemisphere but injections were bilateral. **(d)** Mapping of viral injection sites in the dmPFC (top row of brain slices) and DMS implant sites (bottom row of brain slices) for KO mice expressing either eNpHR3.0 (green x's, N = 13) or eYFP (grey x's, N = 11). Demarcation of viral injection sites for dmPFC is represented by only one x in one hemisphere but injections were bilateral.

Davis et al Supplementary Figure 8

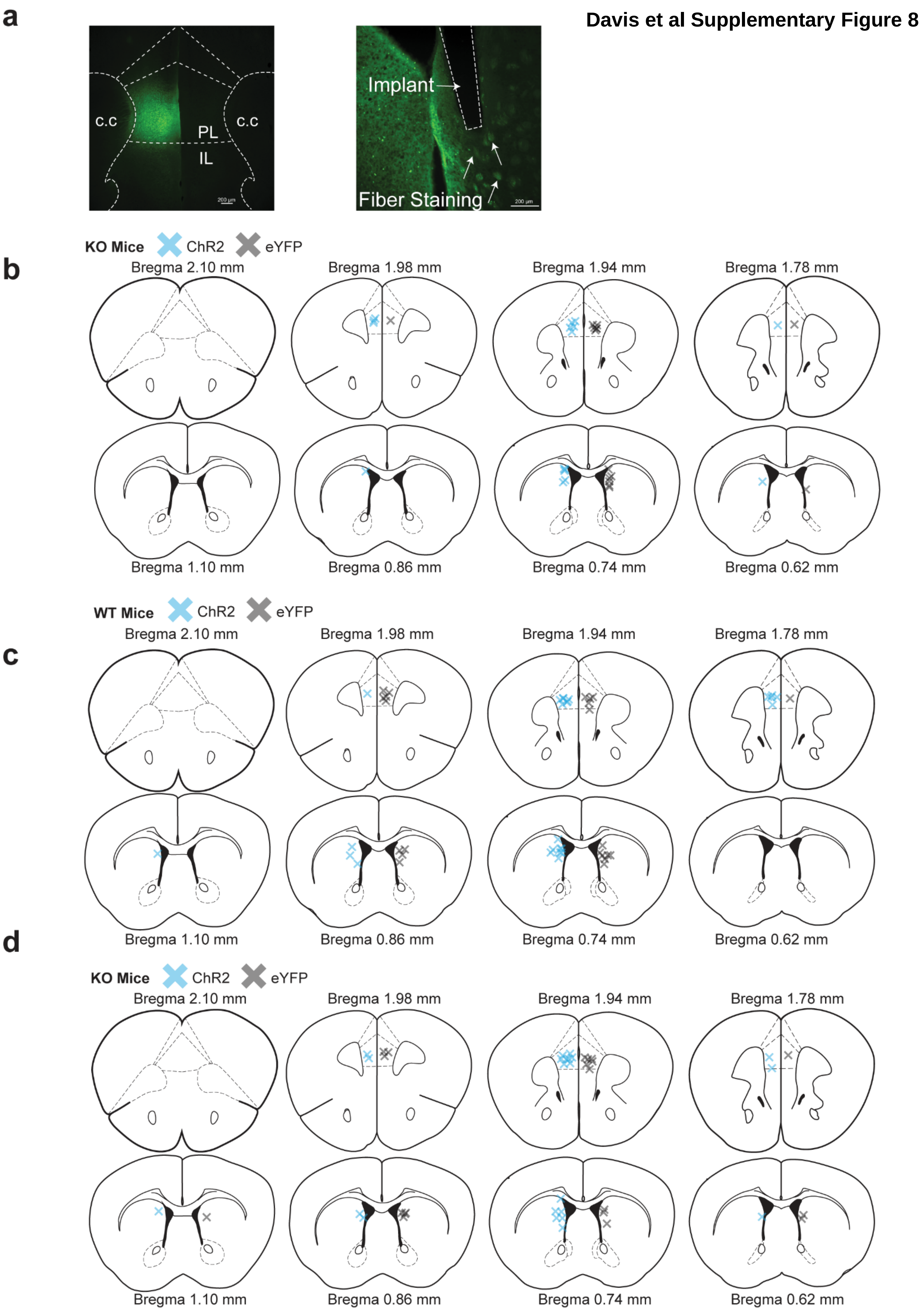

Supplementary Figure 8. ChR2 histology (a) Representative images of AAV5.CaMKIIa.hChR2(H134R)-eYFP viral expression (left) and implant site (right, white arrows highlight fiber staining) (IL = infralimbic cortex, PL = prelimbic cortex, c.c = corpus callosum). **(b)** Mapping of viral injection sites in the dmPFC (top row of brain slices) and DMS implant sites (bottom row of brain slices) for KO mice expressing either ChR2 (blue x's) or eYFP (grey x's) for experiment 1 closed loop stimulation. **(c)** Mapping of viral injection sites in the dmPFC (top row of brain slices) and DMS implant sites (bottom row of brain slices) for WT mice expressing either ChR2 (blue x's) or eYFP (grey x's) for experiment 2 open loop stimulation. **(d)** Mapping of viral injection sites in the dmPFC (top row of brain slices) and DMS implant sites (bottom row of brain slices) for KO mice expressing either ChR2 (blue x's) or eYFP (grey x's) for experiment 2 open loop stimulation.

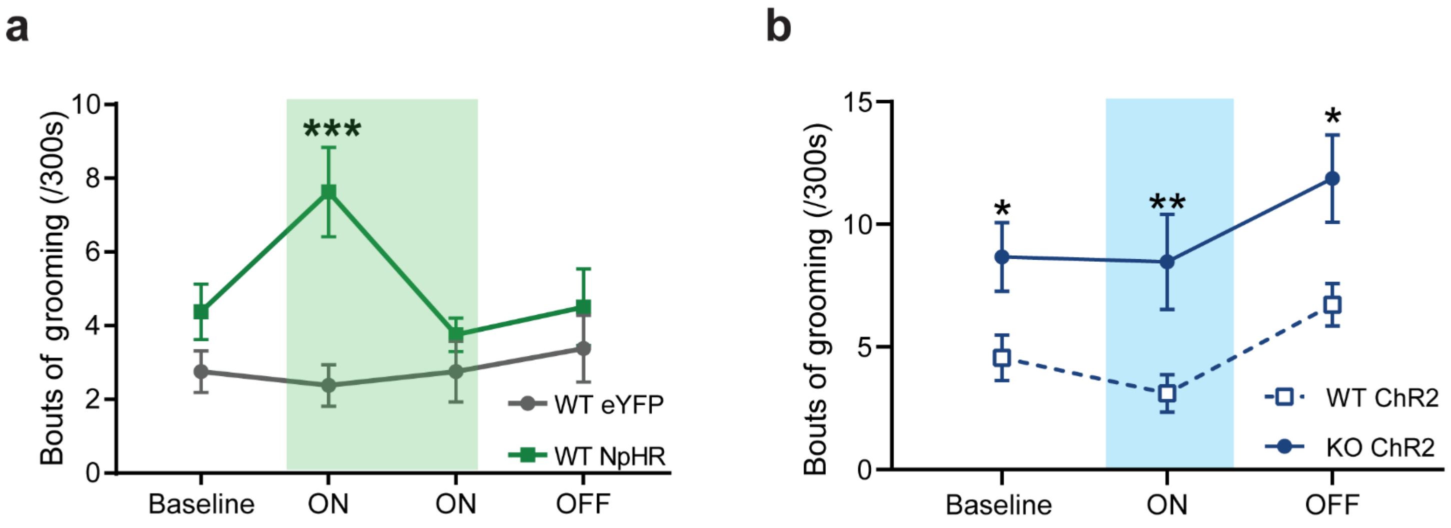

Supplementary Figure 9. Effects of optogenetic manipulation on frequency of grooming behavior. Bar and line graphs show data means +/- SEM. * = P < 0.05, ** = P <0.01, *** = P <0.001 **(a)** Mice expressing NpHR (N = 8) showed increased frequency of grooming during laser on epochs and were significantly different from their eYFP (N = 8) counterparts with a significant main effect of virus and a significant interaction between laser and virus. Multiple comparisons revealed a significant difference between eYFP and NpHR groups during the first 5-minute laser on block (Two-way RM ANOVA: interaction P = 0.0459, df = 3, F = 2.902; laser P = 0.1912, df = 3, F = 1.655; virus group P = 0.0011, df = 1, F = 16.62; subject P = 0.5959, df = 14, F = 14, residual df = 42; Sidak's multiple comparisons: first ON block eYFP vs NpHR P = 0.001). **(b)** SAPAP3 KO mice expressing ChR2 (N = 15) have an elevated frequency of grooming bouts compared to WT mice expressing ChR2 (N = 18) that is not reduced by laser stimulation of dmPFC terminals in the striatum (Two-way RM ANOVA: interaction P = 0.7465, df = 2, F = 0.2938; laser P = 0.0098, df = 2, F = 4.985; genotype P = 0.0008, df = 1, F = 13.77; subject P < 0.0001, df = 31, F = 3.050, residual df = 62; Sidak's multiple comparisons: baseline WT ChR2 vs KO ChR2 P = 0.0294, ON WT ChR2 vs KO ChR2 P = 0.0033, OFF WT ChR2 vs KO ChR2 P = 0.0280).

			Davis et al Supplementary Figure 10		
**Experiment**	**Males**	**Females**	**Age (days)**		
			Mean	Median	Range
**Behavioral Pharmacology**<br>**Saline**<br>**Ketamine**	 4 WTs, 5 KOs<br>8 WTs, 6 KOs	 10 WTs, 6 KOs<br>5 WTs, 6 KOs	328.84	340	154-442
**WT NpHR (exp. 1)**<br>**eYFP**<br>**NpHR**	 2 WTs<br>4 WTs	 6 WTs<br>4 WTs	167.88	168	154-176
**WT NpHR (exp.2)**<br>**eYFP**<br>**NpHR**	 4 WTs<br>8 WTs	 6 WTs<br>10 WTs	208	186	157-396
**KO ChR2 (exp. 1)**<br>**eYFP**<br>**ChR2**	 3 KOs<br>5 KOs	 4 KOs<br>3 KOs	358.47	375	277-398
**WT/KO ChR2 (exp. 2)**<br>**eYFP**<br>**ChR2**	 4 WTs, 4 KOs<br>9 WTs, 7 KOs	 7 WTs, 6 KOs<br>9 WTs, 8 KOs	290.39	234.5	184-486
**KO NpHR + Ketamine**<br>**eYFP**<br>**NpHR**	 5 KOs<br>5 KOs	 6 KOs<br>8 KOs	392.33	375	178-593
**Fiber Photometry**	3 WTs, 3 KOs	8 WTs, 9KOs	268.13	262	173-383

Supplementary Figure 10. Distribution of sex and age across experiments. Kruskal-Wallis test on cohort age $P < 0.0001$. The mean age of mice from the WT NpHR (experiment 1) cohort are statistically different from all other experimental cohorts except for the WT NpHR (experiment 2) cohort (Dunn's multiple comparisons: vs Behavioral Pharmacology cohort $P < 0.0001$, vs Fiber Photometry cohort $P < 0.0001$, vs KO ChR2 (experiment 1) cohort $P < 0.0001$, vs WT and KO ChR2 (experiment 2) cohort $P <0.0001$, vs KO NpHR + Ketamine cohort $P <0.0001$). The mean age of the KO NpHR + Ketamine cohort is statistically different from the WT and KO ChR2 (experiment 2) cohort (Dunn's multiple comparison $P = 0.0298$) and the Fiber Photometry cohort (Dunn's multiple comparison $P = 0.0046$). The mean age of the WT NpHR (experiment 2) cohort is statistically different from the Behavioral Pharmacology cohort (Dunn's multiple comparison $P < 0.0001$), the WT and KO ChR2 (experiment 2) cohort (Dunn's multiple comparison $P = 0.004$), the KO NpHR + Ketamine cohort (Dunn's multiple comparison $P < 0.0001$), and the KO ChR2 (experiment 1) cohort (Dunn's multiple comparison $P < 0.0001$).

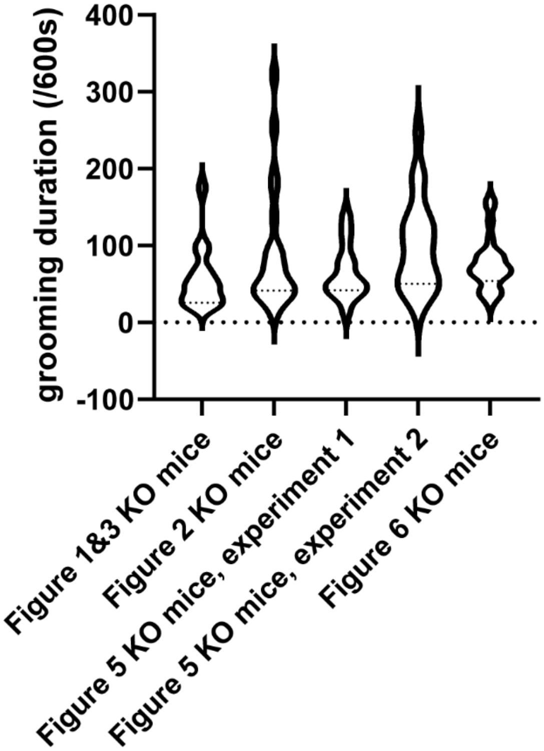

Supplementary Figure 11. Distribution of grooming behavior across experimental cohorts. Kruskal-Wallis test showed no statistical differences in average grooming duration across experimental cohorts (P = 0.0802, Figure 1&3 KO mice = 15 mice, Figure 2 KO mice = 23 mice, Figure 5 KO mice (experiment 1) = 15 mice, Figure 5 KO mice (experiment 2) = 20 mice, Figure 6 KO mice = 24 mice).

Davis et al Supplementary Figure 12

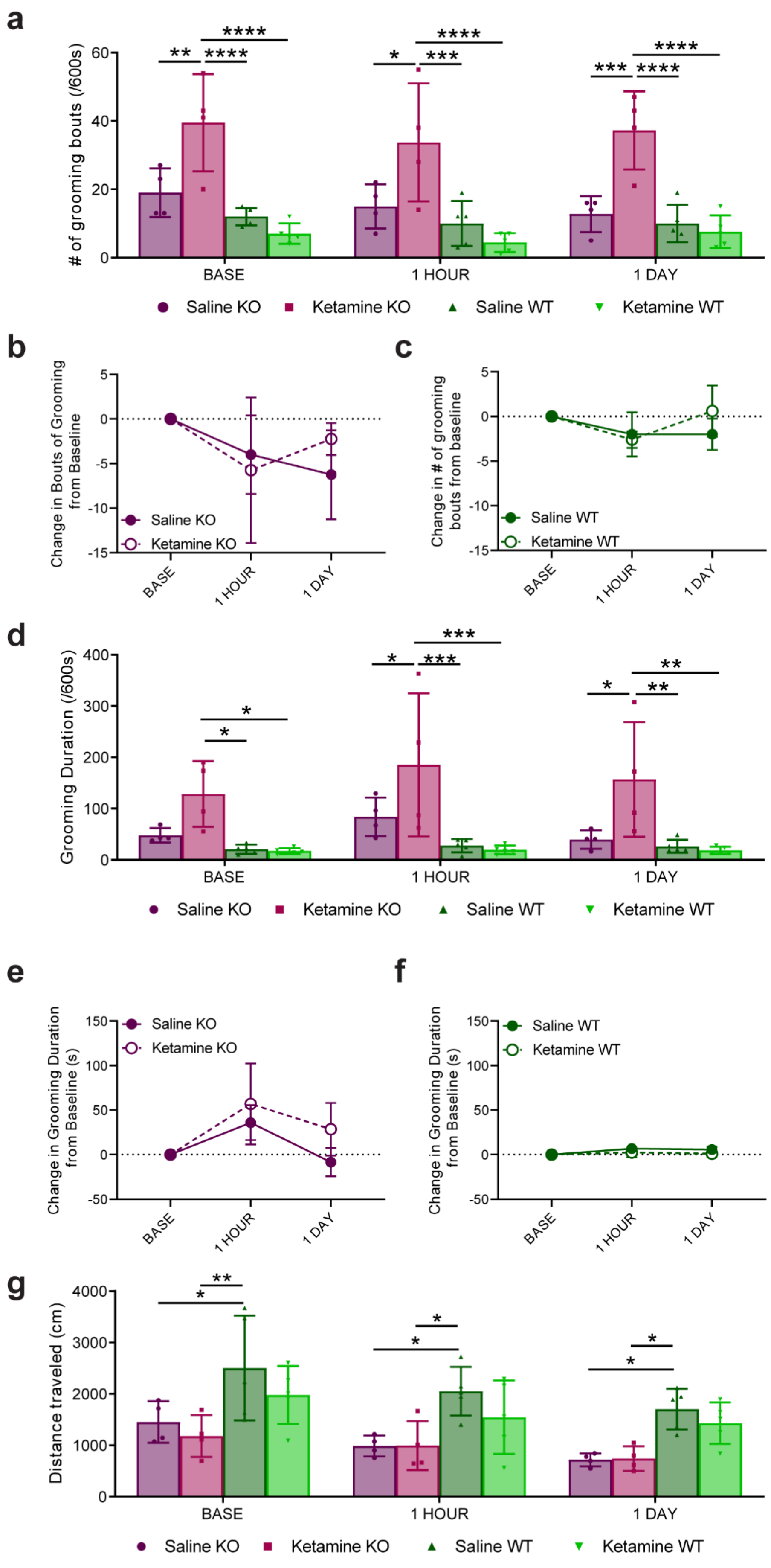

Supplementary Figure 12. Ketamine at a 20mg/kg dose does not reduce grooming behavior. Bar and line graphs show data means +/- SEM. * = $P < 0.05$, ** = $P < 0.01$, *** = $P < 0.001$, **** = $P < 0.0001$. WT-saline = 5 mice, WT-ketamine = 5 mice, KO-saline = 4 mice, KO-saline = 4 mice **(a)** Baseline grooming behavior is quantified in both WT and KO mice. Following saline or ketamine (20 mg/kg) administration grooming measurements are collected 1 hour and 1 day after injection. KO-ketamine mice (purple striped bars) baseline grooming frequency is significantly increased compared to WT controls (green). Ketamine administration has no effect on grooming frequency (Two-Way RM ANOVA: interaction P = 0.8672, df = 6, F = 0.4082; time P = 0.1750, df = 2, F = 1.856; experimental group P < 0.0001, df = 3, F = 17.98; subject P = 0.0008, df = 14, F = 4.036, residual df = 28; Tukey's multiple comparisons: BASE Saline WT vs. Ketamine KO P <0.0001, Ketamine WT vs. Ketamine KO P <0.0001, Saline KO vs. Ketamine KO P = 0.0045; 1 HOUR Saline WT vs. Ketamine KO P = 0.0004, Ketamine WT vs. Ketamine KO P <0.0001, Saline KO vs. Ketamine KO P = 0.0106; 1 DAY Saline WT vs. Ketamine KO P <0.0001, Ketamine WT vs. Ketamine KO P <0.0001, Saline KO vs. Ketamine KO P = 0.0006). **(b)** KO mice given ketamine (open circles) show no difference in grooming frequency from baseline compared to KO mice given saline (filled circles; Two-Way RM ANOVA: interaction P = 0.7556, df = 2, F = 0.2869; time P = 0.4214, df = 2, F = 0.9296; experimental group P = 0.8653, df = 1, F = 0.03167; subject P = 0.1868, df = 6, F = 1.777, residual df = 12). **(c)** WT mice show no significant change in grooming frequency between saline and ketamine groups (Two-Way RM ANOVA: interaction P = 0.5347, df = 2, F = 0.6512; time P = 0.3127, df = 2, F = 1.251; experimental group P = 0.7167, df = 1, F = 0.1413; subject P = 0.0952, df = 8, F = 2.123, residual df = 16). **(d)** KO-ketamine mice have significantly higher levels of baseline grooming but ketamine did not alter grooming duration in KO animals (Two-Way RM ANOVA: interaction P = 0.4880, df = 6, F = 0.9315; time P = 0.0693, df = 2, F = 2.940; experimental group P = 0.0019, df = 3, F = 8.456; subject P < 0.0001, df = 14, F = 5.865, residual df = 28; Tukey's multiple comparisons: BASE Saline WT vs. Ketamine KO P = 0.0199, Ketamine WT vs. Ketamine KO P = 0.0153, Saline KO vs. Ketamine KO P = 0.1502; 1 HOUR Saline WT vs. Ketamine KO P = 0.0003, Ketamine WT vs. Ketamine KO P = 0.0002, Saline KO vs. Ketamine KO P = 0.0444; 1 DAY Saline WT vs. Ketamine KO P = 0.0034, Ketamine WT vs. Ketamine KO P = 0.0017, Saline KO vs. Ketamine KO P = 0.0151). **(e)** KO-ketamine mice show no difference in grooming compared to the KO-saline mice (Two-Way RM ANOVA: interaction P = 0.7565, df = 2, F = 0.2856; time P = 0.1796, df = 2, F = 1.988; experimental group P = 0.3667, df = 1, F =

0.9528; subject P = 0.4825, df = 6, F = 0.9742, residual df = 12). **(f)** WT mice displayed no significant difference in grooming between treatment groups (Two-Way RM ANOVA: interaction P = 0.7045, df = 2, F = 0.3581; time P = 0.3283, df = 2, F = 1.195; experimental group P = 0.4195, df = 1, F = 0.7243; subject P = 0.1172, df = 8, F = 1.976, residual df = 16). **(g)** WT-saline mice move significantly more than KO mice across days (Two-Way RM ANOVA: interaction P = 0.9016, df = 6, F 0.3538; time P < 0.0001, df = 2, F = 15.70; experimental group, P = 0.0067, df = 3, F = 6.190; subject P < 0.0001, df = 14, F = 5.293, residual df = 28; Tukey's multiple comparisons: BASE Saline WT vs. Saline KO P = 0.0244, Saline WT vs. Ketamine KO P = 0.003; 1 HOUR Saline WT vs. Saline KO P = 0.022, Saline WT vs. Ketamine KO P = 0.0234; 1 DAY Saline WT vs. Saline KO P = 0.0382, Saline WT vs. Ketamine KO P = 0.0447).

Supplementary Methods

Supplementary Figure 5 *Ex-Vivo* Electrophysiology Methods: To prepare *ex vivo* slices for whole-cell recordings, mice were deeply anesthetized with isofluorane and transcardially perfused with ice-cold glycerol-based slicing solution, decapitated and the brain was removed. Glycerol-based slicing solution contained (in mM) 250 glycerol, 2.5 KCl, 1.2 NaH2PO4, 10 HEPES, 21 NaHCO3, 5 glucose, 2 MgCl2, 2 CaCl2. Coronal slices 250-300 um thick were made on a vibrating microtome (Leica) while the brain was submerged in cold ACSF containing (in mM) 119 NaCl, 26.2 NaHCO3, 2.5 KCl, 1.3 MgSO4, 2.5 CaCl2, 1 NaH2PO4, 11 Glucose; and constantly bubbled with carbogen (95% O2, 5% CO2). From this moment on, slices were constantly submerged in ACSF bubbled with carbogen. They were left in a recovery chamber at 34C for 45-60 minutes, then stored at RT until the recording time. Pyramidal cells in the dmPFC were targeted visually, using bright field and anatomical landmarks. Cells that were patched and exhibited abnormal properties were discarded (such as abnormal AP shape).

For current clamp recordings, patch electrodes (3-6 MOhm tip resistance) were filled with a Potassium-based internal solution containing (in mM): 130 KMeSO3, 8 NaCl, 2 MgCl2, 0.16 CaCl2, 0.5 EGTA, 10 HEPES, 2 MgATP, 0.3 NaGTP, at 290 mOsm, pH 7.2-7.3. To generate APs, neurons in current clamp were depolarized with a series of 20 000-ms current pulses, with 10 pA between each current pulse. This series of current pulses was alternated with hyperpolarizing steps (−50 pA) to examine input resistance.

Electrophysiological recordings and data acquisition were performed with Multiclamp 700B amplifier and pClamp software (Molecular Devices). Analysis was performed with custom-made Excel (Microsoft) macros and pClamp software (Molecular Devices). Holding current and input resistance were continuously monitored as proxies of recording stability.

Supplementary Figure 6 *Ex-Vivo* Electrophysiology Methods: The below procedures were approved by the Institutional Animal Care and Use Committee of the University of Geneva and by the animal welfare committee of the Canton of Geneva, in accordance with Swiss law.

Virus injections

Using standard stereotactic surgeries under isoflurane anesthesia, Sapap3 KO and WT littermates were injected with AAV5-CamKIIalpha-ChR2(H134R)-eYFP (from UNC vector core) in the dmPFC (300 nl each hemisphere; from bregma and dura mater 1.9 AP, ± 0.35 ML, -1.8 DV).

Patch-clamp electrophysiology

After a minimum of seven weeks of ChR2 expression, mice were injected i.p. with saline (10 ml/kg) or ketamine (30 mg/kg) in their home cages. Mice were sacrificed 24 h after the injections for patch-clamp experiments. Brain slices (220 µm) were cut in oxygenated ice-cold artificial cerebrospinal fluid (aCSF; containing 119 mM NaCl, 11 mM D-glucose, 26.2 mM $NaHCO_3$, 2.5 mM KCl, 1.3 mM $MgCl_2$, 1 mM $NaHPO_4$, 2.5 mM $CaCl_2$), recovered at 31 °C for 15 min and kept at RT until recording. For recordings, brain slices were perfused with oxygenated aCSF at 30 °C containing 100 µM picrotoxin. For strontium experiments, 2 mM $SrCl_2$ replaced $CaCl_2$ in the aCSF. The internal solution consisted of 140 mM potassium gluconate, 10 mM creatine phosphate, 2 mM $MgCl_2$, 4 mM Na_2ATP, 0.3 mM Na_3GTP, 0.2 mM EGTA, 10 mM HEPES, 5 mM KCl. Recording took place in whole-cell voltage-clamp mode with currents amplified (Multiclamp 700B, Axon Instruments), filtered at 2.2 kHz and digitized at 20 kHz (National Instruments Board PCI-MIO-16E4, Igor, Wave Metrics). Cells were held at -70 mV and AMPA receptor currents were evoked with blue light stimuli (470 nm LED; 1 ms pulse duration) every 10 s. For paired-pulse ratio, double pulses with a 50 ms inter-stimulus interval were applied, and 20-30 traces were averaged for quantification. The paired-pulse ratio was calculated as the amplitude of the second pulse divided through the amplitude of the first pulse. For strontium recordings, a single pulse was applied and unitary events were quantified 20-200 ms after stimulation.
