# Supplementary material for: Ketamine increases activity of a fronto-striatal projection that regulates compulsive behavior in SAPAP3 knockout mice
Source: Nat Commun. 2021 Oct 15;12:6040. doi: 10.1038/s41467-021-26247-2 (PMC8519915; doi:10.1038/s41467-021-26247-2)
Supplement: Supplementary file 3 — Reporting Summary [file 41467_2021_26247_MOESM3_ESM.pdf]

nature research

Corresponding author(s): Lisa Gunaydin
Last updated by author(s): Sep 8, 2021

Reporting Summary

Nature Research wishes to improve the reproducibility of the work that we publish. This form provides structure for consistency and transparency in reporting. For further information on Nature Research policies, see our Editorial Policies and the Editorial Policy Checklist.

Statistics

For all statistical analyses, confirm that the following items are present in the figure legend, table legend, main text, or Methods section.

n/a	Confirmed	
☐	☒	The exact sample size (n) for each experimental group/condition, given as a discrete number and unit of measurement
☐	☒	A statement on whether measurements were taken from distinct samples or whether the same sample was measured repeatedly
☐	☒	The statistical test(s) used AND whether they are one- or two-sided<br>*Only common tests should be described solely by name; describe more complex techniques in the Methods section.*
☐	☒	A description of all covariates tested
☐	☒	A description of any assumptions or corrections, such as tests of normality and adjustment for multiple comparisons
☐	☒	A full description of the statistical parameters including central tendency (e.g. means) or other basic estimates (e.g. regression coefficient) AND variation (e.g. standard deviation) or associated estimates of uncertainty (e.g. confidence intervals)
☐	☒	For null hypothesis testing, the test statistic (e.g. F, t, r) with confidence intervals, effect sizes, degrees of freedom and P value noted<br>*Give P values as exact values whenever suitable.*
☒	☐	For Bayesian analysis, information on the choice of priors and Markov chain Monte Carlo settings
☒	☐	For hierarchical and complex designs, identification of the appropriate level for tests and full reporting of outcomes
☒	☐	Estimates of effect sizes (e.g. Cohen's d, Pearson's r), indicating how they were calculated

Our web collection on statistics for biologists contains articles on many of the points above.

Software and code

Policy information about availability of computer code

Data collection	Behavioral data was collected using version 14 of Noldus Ethovision XT software. The fiber photometry data was collected using Tucker-Davis Technologies (TDT) Synapse Suite version 95. Statistical analysis was done using GraphPad Prism 8.
Data analysis	Fiber photometry data was analyzed using code developed in the lab with MATLAB R2017B in conjunction with code developed by Tom Davidson (available on GitHub, https://github.com/tjd2002/tjd-shared-code/tree/master/matlab). Code will be made available on request by emailing the corresponding author of the publication.

For manuscripts utilizing custom algorithms or software that are central to the research but not yet described in published literature, software must be made available to editors and reviewers. We strongly encourage code deposition in a community repository (e.g. GitHub). See the Nature Research guidelines for submitting code & software for further information.

Data

Policy information about availability of data

All manuscripts must include a data availability statement. This statement should provide the following information, where applicable:

- Accession codes, unique identifiers, or web links for publicly available datasets
- A list of figures that have associated raw data
- A description of any restrictions on data availability

Source data is available as an excel file.

Field-specific reporting

Please select the one below that is the best fit for your research. If you are not sure, read the appropriate sections before making your selection.

☒ Life sciences ☐ Behavioural & social sciences ☐ Ecological, evolutionary & environmental sciences

For a reference copy of the document with all sections, see nature.com/documents/nr-reporting-summary-flat.pdf

Life sciences study design

All studies must disclose on these points even when the disclosure is negative.

Sample size	No specific statistical method was used to determine sample size. For the inhibition optogenetic experiments we based our sample size off of preliminary data collected in the lab that showed a significant effect increased grooming with PL-DMS inhibition. The initial experiment design was 10 minutes of PL-DMS laser inhibition, and grooming duration was significantly different between eyfp and nphr groups. Given that the pilot cohort produced significant results, we used similar numbers in a different cohort of animals for the experiment reported in the manuscript. For the stimulation optogenetic experiments, behavioral pharmacology experiments, fiber photometry experiments, and optogenetic-pharmacology combo experiment we used the maximum number of animals we could run in a day for our sample size for the initial performance of these experiments. Since they produced significant effects, we used similar sample sizes to see if we could replicate our results.
Data exclusions	Some data was excluded from the fiber photometry analyses. Prior to analysis of the photometry signals but post-injection, we determined which KO mice were responders versus non-responders to ketamine by calculating a grooming index to assess change in grooming behavior induced by ketamine ((grooming duration post-ketamine – grooming duration post-saline)/(grooming duration post-ketamine + grooming duration post-saline). We then generated the average and standard deviation for WT animals. KO animals had to have a reduction in grooming behavior post-ketamine was at least 1 standard deviation different from the change in grooming behavior of WT animals to be included the fiber photometry analysis. Only 2 out of 15 KO mice did not meet this criterion. Animals were also removed from the study and their data was not analyzed if the the optic implant was incorrectly targeted and so not in the appropriate brain region for the experiment.
Replication	All experiments were performed with at least two separate cohorts of mice to confirm the results, except for experiments performed during the review process. Experiments done during the review process were only done with one cohort and include experiments from figures 5f-h, 6b-d, and 7 . All attempts at replication were successful.
Randomization	The behavioral grooming phenotype of the SAPAP3-KO mice can be quite variable with some animals grooming at levels very similar to their WT litter mates while other KOs are grooming several fold greater than their WT litter mates. As such, we balanced high and low KO groomers across experimental conditions (saline vs ketamine) for the behavioral pharmacology experiments by using preliminary grooming measures. If a cage contained more than one KO or WT animal those animals were placed in separate experimental groups in order to prevent within cage effects. For the optogenetic experiments individual cages contained animals expressing both the control and experimental virus to prevent within cage effects, but which virus the individual mouse was injected with was chosen at random.
Blinding	For all experiments those scoring grooming behavior were blind to genotype and where applicable, drug administration (ketamine or saline), and virus expression (NpHR, ChR2, eYFP).

Reporting for specific materials, systems and methods

We require information from authors about some types of materials, experimental systems and methods used in many studies. Here, indicate whether each material, system or method listed is relevant to your study. If you are not sure if a list item applies to your research, read the appropriate section before selecting a response.

Materials & experimental systems

n/a	Involved in the study
☒	☐ Antibodies
☒	☐ Eukaryotic cell lines
☒	☐ Palaeontology and archaeology
☐	☒ Animals and other organisms
☒	☐ Human research participants
☒	☐ Clinical data
☒	☐ Dual use research of concern

Methods

n/a	Involved in the study
☒	☐ ChIP-seq
☒	☐ Flow cytometry
☒	☐ MRI-based neuroimaging

Animals and other organisms

Policy information about studies involving animals; ARRIVE guidelines recommended for reporting animal research

Laboratory animals	All mice were on a C57BL/6J background strain. All animals were at least 120 days before being used to allow for the SAPAP3-KO grooming phentoype to emerge (age-dependent phenotype). Both males and females were used. All mice were raised in normal light

	conditions (12:12 light/dark cycle), with ad libitum access to food and water. Mouse colony rooms were kept at an ambient temperature of 69-73 degrees Fahrenheit with 50-65% humidity.
Wild animals	The study did not involve wild animals.
Field-collected samples	The study did not involve field samples.
Ethics oversight	Animal protocol was approved by UCSF's IACUC committee.

Note that full information on the approval of the study protocol must also be provided in the manuscript.
